# The Diterpenoid 7-Keto-Sempervirol, Derived from *Lycium chinense*, Displays Anthelmintic Activity against both *Schistosoma mansoni* and *Fasciola hepatica*

**DOI:** 10.1371/journal.pntd.0003604

**Published:** 2015-03-13

**Authors:** Jennifer Edwards, Martha Brown, Emily Peak, Barbara Bartholomew, Robert J. Nash, Karl F. Hoffmann

**Affiliations:** 1 Institute of Biological, Environmental and Rural Sciences (IBERS), Aberystwyth University, Aberystwyth, United Kingdom; 2 Phytoquest Ltd, Plas Gogerddan, Aberystwyth, United Kingdom; McGill University, CANADA

## Abstract

**Background:**

Two platyhelminths of biomedical and commercial significance are *Schistosoma mansoni* (blood fluke) and *Fasciola hepatica* (liver fluke). These related trematodes are responsible for the chronic neglected tropical diseases schistosomiasis and fascioliasis, respectively. As no vaccine is currently available for anti-flukicidal immunoprophylaxis, current treatment is mediated by mono-chemical chemotherapy in the form of mass drug administration (MDA) (praziquantel for schistosomiasis) or drenching (triclabendazole for fascioliasis) programmes. This overreliance on single chemotherapeutic classes has dramatically limited the number of novel chemical entities entering anthelmintic drug discovery pipelines, raising significant concerns for the future of sustainable blood and liver fluke control.

**Methodology/ Principle Findings:**

Here we demonstrate that 7-keto-sempervirol, a diterpenoid isolated from *Lycium chinense*, has dual anthelmintic activity against related *S*. *mansoni* and *F*. *hepatica* trematodes. Using a microtiter plate-based helminth fluorescent bioassay (HFB), this activity is specific (Therapeutic index = 4.2, when compared to HepG2 cell lines) and moderately potent (LD_50_ = 19.1 μM) against *S*. *mansoni* schistosomula cultured *in vitro*. This anti-schistosomula effect translates into activity against both adult male and female schistosomes cultured *in vitro* where 7-keto-sempervirol negatively affects motility/behaviour, surface architecture (inducing tegumental holes, tubercle swelling and spine loss/shortening), oviposition rates and egg morphology. As assessed by the HFB and microscopic phenotypic scoring matrices, 7-keto-sempervirol also effectively kills *in vitro* cultured *F*. *hepatica* newly excysted juveniles (NEJs, LD_50_ = 17.7 μM). Scanning electron microscopy (SEM) evaluation of adult *F*. *hepatica* liver flukes co-cultured *in vitro* with 7-keto-sempervirol additionally demonstrates phenotypic abnormalities including breaches in tegumental integrity and spine loss.

**Conclusions/ Significance:**

7-keto-sempervirol negatively affects the viability and phenotype of two related pathogenic trematodes responsible for significant human and animal infectious diseases. This plant-derived, natural product is also active against both larval and adult developmental forms. As such, the data collectively indicate that 7-keto-sempervirol is an important starting point for anthelmintic drug development. Medicinal chemistry optimisation of more potent 7-keto-sempervirol analogues could lead to the identification of novel chemical entities useful for future combinatorial or replacement anthelmintic control.

## Author Summary

Schistosomiasis and fascioliasis are caused by two related trematodes found within the phylum Platyhelminthes (flatworms), and are classified as neglected diseases of poverty due to their effects on people living in the most underprivileged areas of the world. With no vaccine currently near development, and the existing strategy for global control based on the over-reliance on single-class chemotherapies, there is an urgent requirement for the identification of next generation anthelmintics. Here we demonstrate that 7-keto-sempervirol, a natural product derived from *Lycium chinense*, displays dual anthelmintic activity towards both *Schistosoma mansoni* (causative agent of schistosomiasis) and *Fasciola hepatica* (causative agent of fascioliasis). Utilising objective and phenotypic matrices, we show this activity to be selective (compared to a human cell line) and moderately potent against *S*. *mansoni* and *F*. *hepatica* larvae. This anti-larval effect translates into additional activity against both *S*. *mansoni* and *F*. *hepatica* adults where 7-keto-sempervirol induces phenotypic abnormalities including tegumental damage, motility disruption and oviposition inhibition. Due to 7-keto-sempervirol’s anthelmintic activity against multiple life stages of two parasitic trematodes, we contend that this starting chemical scaffold could be used to develop more effective compounds useful in controlling important parasites of biomedical and commercial relevance.

## Introduction

Schistosomiasis and fascioliasis are Neglected Tropical Diseases (NTDs) caused by related parasitic blood (*Schistosoma* sp. including *Schistosoma mansoni*) and liver (*Fasciola* sp including *Fasciola hepatica*) flukes found within the phylum Platyhelminthes. These NTDs are responsible for chronic conditions of biomedical and veterinary significance and collectively affect a considerable proportion of the world’s human and animal populations. Globally, approximately 200 million people are currently afflicted by schistosomiasis, with this chronic disease being most prominent in tropical/subtropical regions of poverty-stricken, rural areas [1]. While fascioliasis is one of the most important parasitic diseases of ruminant livestock animals, it also negatively impacts 2.4 to 17 million humans worldwide by inducing chronic liver pathologies in infected individuals [2].

Control of these two NTDs remains largely centred on the use of large-scale chemotherapy administered to infected individuals in high prevalence areas. Praziquantel (PZQ) is presently the gold standard drug of choice for schistosomiasis control due to its safety, low cost, and activity towards the adult life stage of the three major, human-infective species (*S*. *mansoni*, *Schistosoma haematobium* and *Schistosoma japonicum*) [3]. For fascioliasis chemotherapy, triclabendazole (TCBZ) remains the drug of choice and is the only available compound effective against both adult and juvenile liver fluke lifecycle stages [4]. In both cases, the over-reliance on a single drug class for maintaining the future of blood and liver fluke control has generated significant concerns that drug resistant flukes could eventually develop (*Schistosoma*) or increase in prevalence (*Fasciola*). Although potential new replacement or combinatorial anthelmintic compounds have recently been identified [5–11], concerns have been raised regarding the insufficient activity in this research area due to the perceived sustainability of current options [12,13].

Moving forward, it is becoming evident that a relatively untapped source of chemical entities for developing new anthelmintics is plants and their natural products [14–16]. Plants possess a diverse arsenal of chemical reserves that have evolved to aid in plant protection and competition, and this has been exploited for medicinal uses for centuries [17]. For example, while artemisinin (derived from *Artemisia annua*) and derivatives represent the current, frontline anti-malarials [18], they also display significant activity against *Schistosoma* and *Fasciola* [7,19]. Further interrogation of the chemical diversity found in plants suggests that terpenoids (terpenes) are the most abundant and numerous of the plant secondary products and they possess features, including considerable structural variation [20], that may be exploitable as next-generation anthelmintics. In clear support of this, previous studies have demonstrated that topical terpenoid application to the skin effectively prevents schistosome penetration, providing an innovative chemoprophylactic method to avert schistosomiasis [21,22]. However, it is presently unclear how terpenoids affect schistosome biology or, indeed, if they have activity against other parasitic trematodes, including *F*. *hepatica*, due to lack of detailed investigations.

In this study a diterpenoid (7-keto-sempervirol) extracted and purified from *Lycium chinense* (common name Wolfberry), a plant traditionally used in Asian medicine since 1000 AD [23], was thoroughly investigated for its anthelmintic properties against *S*. *mansoni* and *F*. *hepatica* larvae and adults. Here, we demonstrate that 7-keto-sempervirol selectively kills larval stages of each trematode and induces tegumental damage, as well as motility disruption, in both hermaphroditic and dioecious adults. Furthermore, 7-keto-sempervirol dramatically inhibits the developmental maturation and oviposition of phenotypically normal *S*. *mansoni* eggs. Collectively these findings suggest that diterpenoids, such as 7-keto-sempervirol, have broad activities against related trematodes and should be further investigated as starting points for combating both schistosomiasis and fascioliasis.

## Methods

### Ethics statement

All procedures performed on mice adhered to the United Kingdom Home Office Animals (Scientific Procedures) Act of 1986 (project license PPL 40/3700) as well as the European Union Animals Directive 2010/63/EU and were approved by Aberystwyth University’s (AU) Animal Welfare and Ethical Review Body (AWERB).

### Compound storage and handling

The diterpenoid 7-keto-sempervirol was isolated from the root of the temperate plant *Lycium chinense* (The Organic Herb Trading Company, Milverton, UK). Two kilograms of ground material was extracted in CH_2_Cl_2_ using a Soxhlet system. Nine fractions were obtained using Biotage 75 flash chromatography (silica gel, eluted with a step gradient of increasing polarity: n-hexane—ethylacetate—methanol). 7-keto-sempervirol was isolated from these fractions via reverse-phase preparative HPLC (C18 preparative column, eluted with a gradient- water: acetonitrile: 0.1% trifluroacetic acid in acetonitrile, with UV detection). The compound structure was then determined by UV, ^1^H NMR, ^13^C NMR and LC-MS analyses and also through direct comparison with the literature. The compound auranofin (Sigma-Aldrich, UK), a thioredoxin glutathione reductase (TGR) inhibitor capable of killing schistosomes, was used at 70 μM (final concentration) [24] as a dead control for the Helminth Fluorescent Bioassay (HFB) [25]. Both compounds (auranofin and 7-keto-sempervirol) were stored at -80°C in DMSO until use.

### *Schistosoma mansoni* schistosomula culture

*S*. *mansoni* (NRMI strain) cercariae were shed from infected *Biomphalaria glabrata* snails (NMRI strain) by exposure to 2 hours of light in an artificially heated room (26°C). Collected cercariae were mechanically transformed into schistosomula as previously described [26] and resuspended in culture media comprising DMEM (Dulbecco’s Modified Eagle Medium, Sigma-Aldrich, UK) lacking phenol red, but containing 4.5 g/l glucose, 2 mM L-glutamine, 200 U/ml penicillin and 200 μg/ml streptomycin (all Sigma-Aldrich, UK). Schistosomula were then transferred to a black sided, flat-bottom (optically clear) 96-well microtiter plate (Star Lab, UK) at a density of 1000 parasites per 100 μl well. The plate was then incubated at 37°C and 5% CO_2_ for 24 hr to allow parasite equilibration.

### Adult *Schistosoma mansoni* culture

*S*. *mansoni* adult parasites were recovered by hepatic portal perfusion from Tuck Ordinary mice (Harlan Laboratories, UK) experimentally infected seven weeks earlier with 200 cercariae. Washed adult worms were cultured in DMEM containing phenol red, 4.5 g/l glucose, supplemented with 10% foetal calf serum, 2 mM L-glutamine, 200 U/ml penicillin, 200 μg/ml streptomycin (all Sigma-Aldrich, UK). Schistosome cultures were maintained at 37°C in a humidified atmosphere containing 5% CO_2_ for 24 hr prior to further manipulations. For egg laying experiments, five adult worm pairs per ml of culture medium (48-well tissue culture plates) were cultivated as above for a total of 72 hr (in the presence/absence of 7-keto-sempervirol). Eggs were counted after 72 hr and classified as normal (oval and containing a fully-formed lateral spine) or abnormal (lacking an oval shape and fully formed lateral spine).

### *Fasciola hepatica* newly excysted juvenile (NEJ) culture

*F*. *hepatica* metacercariae (Baldwin Aquatic, Inc., OR, USA) were transformed into newly excysted juveniles (NEJs) and equilibrated for 4 hr in *Fasciola* saline according to established methodologies [27]. After equilibration, NEJs were distributed into black sided, flat-bottom (optically clear) 96-well microtiter plates (Star Lab) at a density of 100 parasites per 100 μL well and cultured at 37°C in a humidified atmosphere containing 5% CO_2_ subject to further treatments.

### Adult *Fasciola hepatica* culture

*F*. *hepatica* adult flukes were recovered from sheep livers (Ridgeway Research, UK), washed and cultured in media comprising DMEM (lacking phenol red) containing 4.5 g/l glucose (Sigma, UK). This was supplemented with 2.2 mM Ca(C_2_H_3_O_2_)_2_, 2.7 mM MgSO_4_, 61 mM glucose, 15 mM HEPES, 1 μM serotonin and 5 μg/ml gentamycin (all Sigma, UK). Adult fluke were placed in 12-well tissue culture plates, 1 parasite per well in 1 ml of culture media and cultivated for 48 hr at 37°C in a humidified atmosphere containing 5% CO_2_.

### HepG2 cell culture and CellTitre-Glo assay

The human HepG2 cell line purchased from the European collection of cell cultures (ECACC 85011430) was grown to confluency in culture media comprising EMEM (Eagle’s Minimum Essential Medium) supplemented with 10% bovine calf serum, 2 mM L-glutamine, 1% non-essential amino acid solution (all Sigma Aldrich, UK) and 100 U/ml penicillin and 100 μg/ml streptomycin (Invitrogen, UK). Cells were resuspended by trypsinisation (0.25% v/v for 5 min), rinsed and distributed into black sided, flat-bottom (optically clear) 96-well microtiter plates (StarLab, UK) at 50 μl/well (1x10^5^ cells/ well). The plate was equilibrated in a humidified atmosphere containing 5% CO_2_ at 37°C for 2 hrs and then test compounds were added to relevant wells to create a total well volume of 100 μl/well. Live controls included cells incubated in the same concentration (1% v/v) of DMSO that was used in the experimental (compound treated) wells. Dead controls included cells incubated with 1% v/v Triton X-100 (Sigma-Aldrich) [28]. Tissue culture plates were returned to a humidified atmosphere containing 5% CO_2_ at 37°C for a further 24 hrs.

The CellTitre-Glo reagents were prepared according to the manufacturer’s instructions (Promega, UK). Tissue culture plates were equilibrated to RT for 30 min before CellTitre-Glo reagents were distributed into each plate well at 100 μl/well (total volume 200 μl/well) and mixed on an orbital shaker for 2 min. Cells were then stabilised for 10 min at RT and bubbles that may have formed were removed using a sterilised needle. After this time luminescent signal was read utilising the BMG Labtech Polarstar Omega Plate Reader and exported into Microsoft Excel for further analysis and conversion into percentage viability.

### Helminth Fluorescent Bioassay (HFB)

The HFB was utilised to objectively determine the viability of schistosomula and NEJ parasites co-cultured in the presence of 7-keto-sempervirol, auranofin (70 μM) [8,29], DMSO (1% v/v) or media only. HFB methodology applied to schistosomula was performed as previously described [25] with a minor alteration. Here, as indicated in the schistosomula culture methods, parasite viability was measured in the absence of foetal calf serum (FCS). A slight modification of the HFB was also applied to NEJs and included assaying only 100 parasites/well (as opposed to 1000 schistosomula/well). A total of 100 μl of test substance (varying concentrations) was added to each well containing 100 μl of suspended parasites and cultured for 24 hr in a humidified atmosphere at 37°C before the HFB was performed.

### Phenotypic measurements

The *in vitro* activity of 7-keto-sempervirol on *S*. *mansoni* adult worms and *F*. *hepatica* NEJs was assessed by measuring motility disturbances and morphological variations in comparison to an appropriate live control (DMSO, 1% v/v). The scoring matrix used to assess *S*. *mansoni* adult worm viability was based on the standard operating procedure for compound screening at the Special Programme for Research and Training in Tropical Diseases, World Health Organization, WHO-TDR as previously described [30]. Motility was numerically scored from 0–4 with 0 being total absence of all motility, 1 indicating absence of motility other than gut peristalsis, 2 representing minimal activity such as occasional head and tail movement, 3 demonstrating slow activity and 4 signifying normal activity. Morphological descriptors of the parasite were also recorded during phenotypic assessment and included ‘knots’ developing along the normally cylindrical body line of the adult worms and the sloughing, blebbing or tubercle swelling of the tegument. Scoring matrix assessment of the worms was performed at 24, 48 and 72 hr post treatment.

NEJ phenotyping (a supportive metric of the HFB) was performed using values derived from both movement indices and morphologic formats as previously described [31,32]. The movement score ranges from 1–5 with 1 representing good/normal movement and 5 representing a complete absence of movement. The morphologic score ranges from 1–6 with 1 representing a good/normal phenotype and 6 signifying a severely dissolved/granulated parasite. This scoring matrix assessment (values derived from summation of both movement and morphology metrics) was conducted at 24 hr post treatment.

### Scanning electron, laser confocal scanning and high content imaging microscopy

Prior to scanning electron microscopy (SEM) analysis, adult schistosomes and liver flukes were first fixed in 2.5% (w/v) gluteraldehyde in phosphate buffered saline (PBS) for 24 hr at 22–24°C. After fixation, worms were washed 3 times in 1 X PBS, pH 7.5 and stored in the same buffer at 4°C until use [33]. Fixed parasite material was subsequently washed twice in double distilled water and dehydrated in ascending acetone percentages (30, 50, 70, 80, 90, 95, and 100%) for 15 minutes each as previously described [33,34]. Dehydrated worms were then critically dried (Polaron Critical Point Dryer E3000) for 1 hr in 100% acetone (critical point of CO_2_ is 7.38 MPa at a temperature of 31°C). Critically dried worms were mounted on aluminium stubs, sputter coated with platinum/palladium and observed under a Hitachi S-4700 field emission scanning electron microscope [34].

For laser scanning confocal microscopy (LSCM), adult schistosomes were first fixed in a solution containing 2% (v/v) acetic acid, 25% (v/v) formalin, 48% (v/v) ethanol and 25% (v/v) H_2_O at room temperature for 24 hr. After fixation, worms were stained with Langeron’s Carmine as previously described [35]. Worms were then mounted on glass microscope slides in DPX (distyrene, plasticiser, xylene) as previously described [36] and observed using a Leica TCS SP5II laser scanning confocal microscope, equipped with a 40X oil immersion objective and a 488 nm Argon laser and a 561nm DPSS laser.

*S*. *mansoni* eggs laid by adult females after 72 hr *in vitro* cultures (+/- 7-keto-sempervirol) were fixed in a 10% formaldehyde solution to prepare the biological tissue for microscopy. Fixed eggs were then phenotypically assessed by an ImageXpress micro XL High Content Imager (Molecular Devices, UK). Fluorescent images were obtained using a FITC filter (40x magnification). Schistosomula subjected to the HFB were visualised on the ImageXpress micro XL High Content Imager using FITC (to identify fluorescein diacetate positive parasites) and TRITC (to identify propidium iodide positive parasites) filters (10X magnification).

### Statistical analysis

A One Way ANOVA was utilised to identify any significant differences between more than three treatment groups followed by *post hoc* testing with the Tukey’s test to identify significantly different means (means that do not share a letter are significantly different). Student’s t-test was utilised to determine significant differences between two treatment groups.

## Results

### Anti-schistosomal 7-keto-sempervirol properties

**Fig 1:**
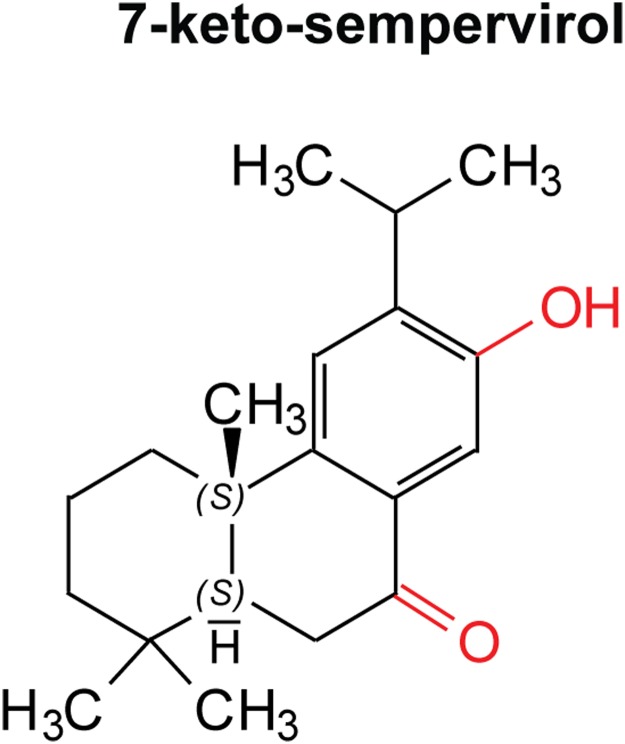
7-keto-sempervirol is a diterpenoid phenol derived from *Lycium chinense*. This compound possesses a diterpenoid (C_20_) (4 isoprene units) scaffold consisting of a single phenyl group, one hydroxyl group (red) and one carbonyl group (red). In light of Lipinski’s rule of 5 (RO5) [57], this compound only possesses one violation to the rule (log p > 5), however, three out of the four core rules are satisfied.

**Fig 2:**
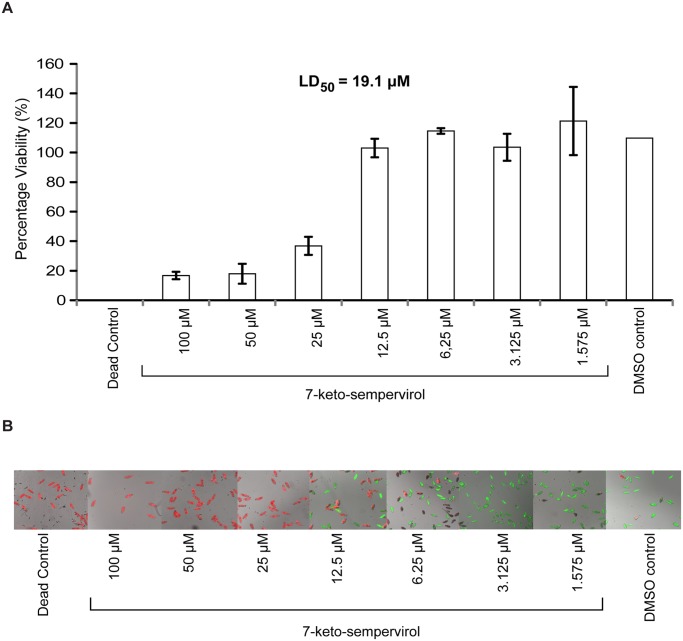
The diterpenoid 7-keto-sempervirol displays lethal activity against *Schistosoma mansoni* schistosomula. Schistosomula (96 well plate format; 1000 parasites/well; triplicate wells for each concentration assessed) were co-cultivated with a 50% dilution series of 7-keto-sempervirol (100, 50, 25, 12.5, 6.25, 3.125, 1.563 μM) at 37°C and 5% CO_2_ for 24 hr and subjected to the helminth fluorescent bioassay (HFB). A) Fluorescent data derived from wells were converted into corresponding Log_10_ values and the mean percentage viability was transformed into probit values to create a dose-response curve as previously described [25]. An LD_50_ of 19.1 μM was calculated from this dose-response curve. Error bars represent the standard deviation of the mean (SD). B) High content imaging of fluorescently labelled parasites (fluorescein diacetate, green and propidium iodide, red) co-cultured with titrated 7-keto-sempervirol supports the HFB measurements. The concentration of fluorophores employed for high content imaging were the same as previously described for the HFB [25]. Dead control = parasites co-cultured with 70 μM auranofin. DMSO control = parasites co-cultured with 1% (v/v) DMSO. Parasites were imaged at 10 x magnification on a high content imaging system processed by the MetaXpress version 5.10.46 software utilising both FITC and TRITC filters.

Due to the previously described action of select terpenes on schistosome viability [21,22] and definitive host penetration [37], we became interested in assessing the potential anthelmintic activity of 7-keto-sempervirol, a diterpenoid purified from *Lycium chinense* (Fig. 1). Employing the HFB [25], a titration series of 7-keto-sempervirol (100 μM–1.575 μM) was first used to assess the ability of this diterpenoid to affect schistosomula viability during *in vitro* co-culture (Fig. 2). At high 7-keto-sempervirol concentrations (100–25 μM), almost all of the schistosomula were killed or severely affected as indicated by low percent viability values (Fig. 2A) and fluorescent microscopic images of individual parasites (PI positive parasites > FDA positive parasites) (Fig. 2B). Lower 7-keto-sempervirol concentrations (between 6.25 μM–1.575 μM) had little effect on schistosomula viability and phenotype when compared to the DMSO control parasites. Based on these titration experiments, an LD_50_ of 19.1 μM was derived for 7-keto-sempervirol on the schistosomula lifecycle stage. A parallel set of titration experiments was additionally performed with the human HepG2 cell line (S1 Fig.) and demonstrated a 7-keto-sempervirol derived LD_50_ of 80 μM. 7-keto-sempervirol, therefore, demonstrates a therapeutic index of 4.2 towards the intra-mammalian schistosomula lifecycle stage.

**Fig 3:**
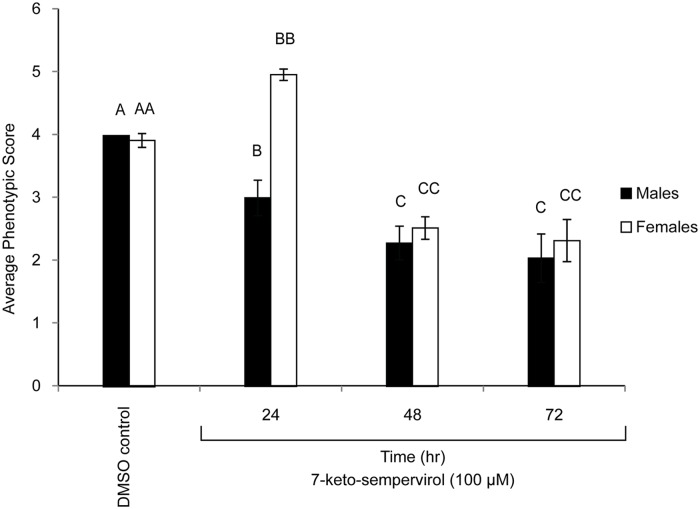
Adult *Schistosoma mansoni* motility is reduced in the presence of 7-keto-sempervirol. World Health Organisation motility metrics were used for scoring adult *S*. *mansoni* male and female worm phenotypes (5 worm pairs/ml) co-cultured (37°C and 5% CO_2_) with 7-keto-sempervirol (100 μM final concentration) for 24 hr, 48 hr and 72 hr. Phenotypes derived from a control group (media containing 1% v/v DMSO) of male and female worms cultivated for 24 hr (worm pairs cultured for 48 hr or 72 hr were identical to the 24 hr worms, S2 Fig.) is also illustrated. Mean motility values are indicated as histograms (n = 5 replicates/time-point) and the error bars represent the standard deviation of the mean (SD). Different letters indicate a significant difference between the mean phenotypic scores calculated by ANOVA and Tukey’s *post-hoc* tests.

**Fig 4:**
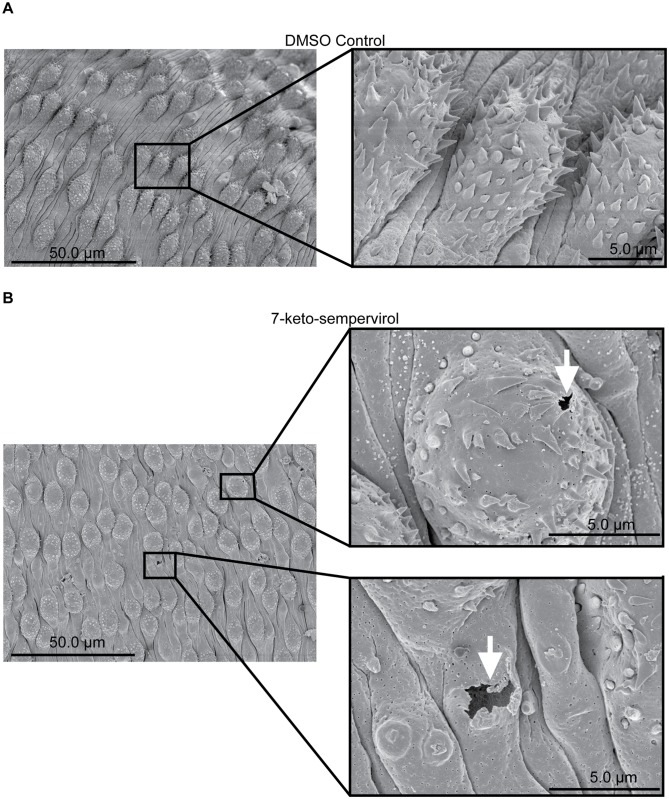
The diterpenoid 7-keto-sempervirol induces surface tegumental damage in adult *Schistosoma mansoni* males. Scanning Electron Microscopic (SEM) images of adult *S*. *mansoni* male worms cultured for 72 hr at 37°C and 5% CO_2_ in the presence (100 μM) or absence (1% v/v DMSO) of 7-keto-sempervirol. A) (left) 1.00K x magnification of a control (1% v/v DMSO) adult male surface (anterior end) and corresponding (right) 6.00K x magnification of normal tubercles. B) (left) 1.00K x magnification of a 7-keto-sempervirol treated (100 μM), adult male surface (anterior end) and enlarged (right) 9.00K x magnification of two indicated (black boxes) areas of tegumental damage. Holes in the surface tegument/tubercles are indicated by white arrows. These images are representative of 5 adult worms/condition.

Based on its anti-schistosomula activity, 7-keto-sempervirol’s ability to affect adult schistosome motility, surface-tegument morphology and egg development was subsequently measured using both WHO-adopted indices and microscopic measures. Here, 7-keto-semperivol displayed a significant effect on both male and female worm motility at the highest concentration used in this study (100 μM) at 24 hr and 48 hr post treatment compared to the DMSO (24 hr) control group (Fig. 3). For both genders, there was no significant difference between 48 hr and 72 hr treatments at this concentration (Fig. 3) nor in DMSO treated worms cultivated for 48 hr or 72 hr (S2 Fig.). Interestingly, female worms (unlike males) displayed a 7-keto-semperivol-induced hyperactivity at 24 hr post-treatment (Fig. 3). There were motility and phenotypic discrepancies observed for some individuals cultured in the presence of 10 μM 7-keto-sempervirol, but these differences were not significantly different compared to the DMSO control group (S3 Fig.). Scanning electron microscopy (SEM) of adult male worms co-cultured in the presence of 7-keto-sempervirol (100 μM for 72 hr) further revealed tubercle swelling, spine loss/shortening and surface holes across the tegument (Fig. 4).

**Fig 5:**
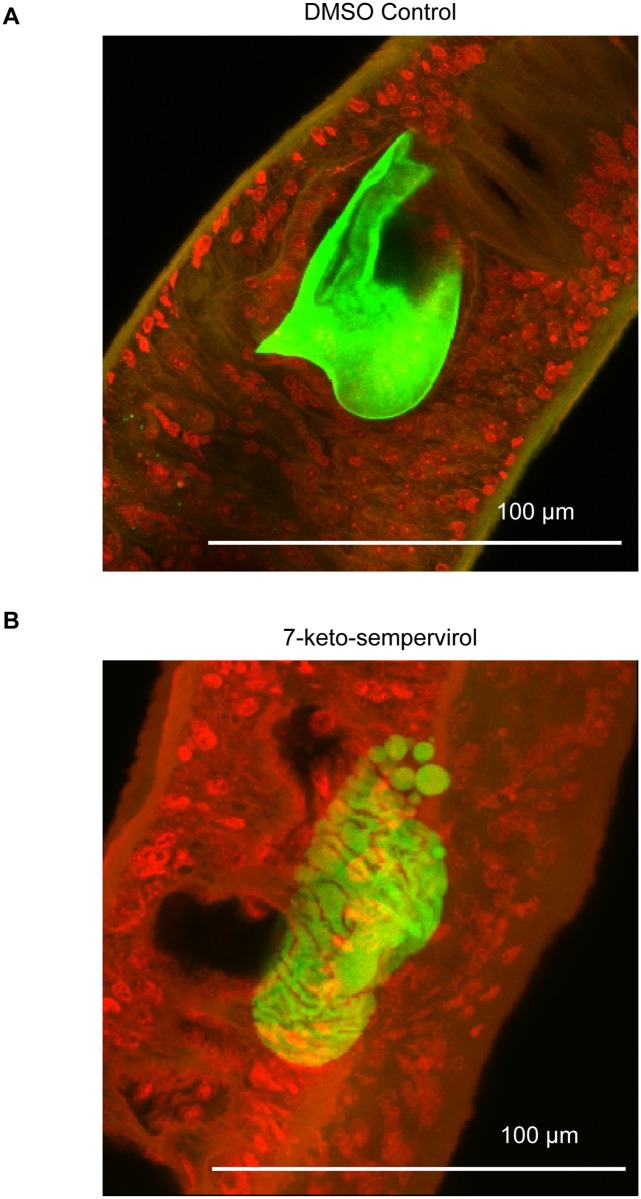
*Schistosoma mansoni* egg-shell formation is inhibited by 7-keto-sempervirol. Adult *S*. *mansoni* female worms were cultured (+/- 100 μM 7-keto-sempervirol) for 24 hr at 37°C and 5% CO_2_. A) 40x laser scanning confocal microscopic image of an egg derived from a control female (1% v/v DMSO) demonstrating regular auto-fluorescence and a well-formed lateral spine (normal architecture of a *S*. *mansoni* egg). B) 40x laser scanning confocal microscopic image of an egg derived from one adult female worm treated with 100 μM 7-keto-sempervirol. Images are representative of typical *in utero* egg phenotypes obtained from 5 worms/condition.

**Fig 6:**
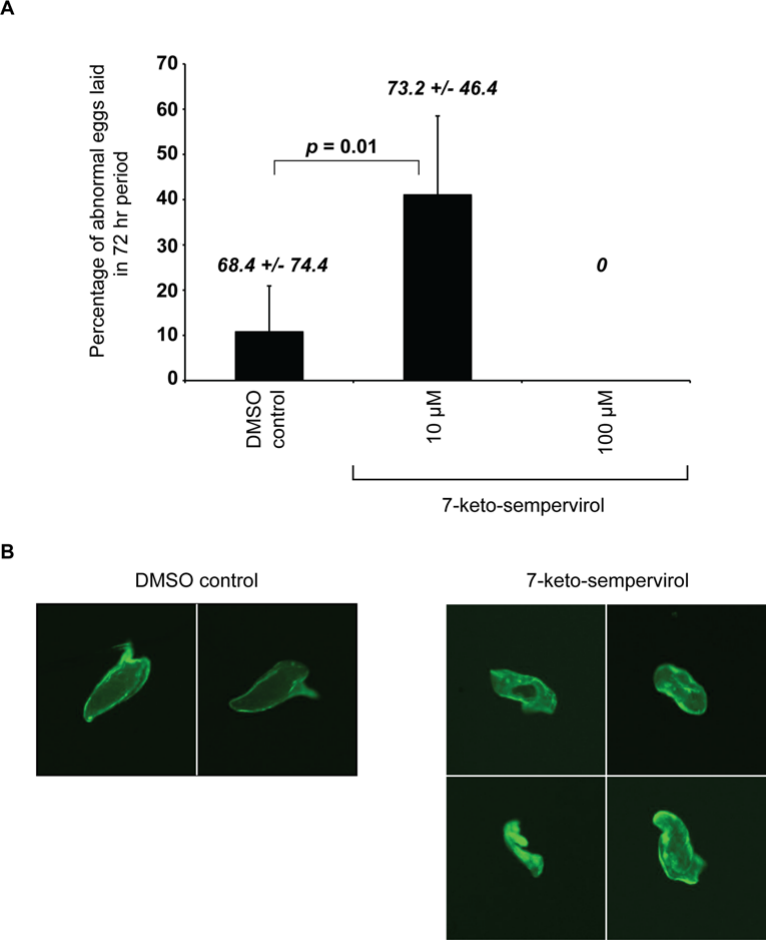
Schistosoma mansoni oviposition is inhibited by 7-keto-sempervirol. Adult worm pairs (5 pairs/ml) were co-cultured (37 ^**o**^C and 5% CO_2_) with 1% v/v DMSO (DMSO control), 10 μM 7-keto-sempervirol or 100 μM 7-keto-sempervirol for 72 hr. A) Bar charts represent the mean percentage of abnormal eggs (lacking a fully formed lateral spine) produced after 72 hr (n = 5 replicates/treatment). Error bars represent the standard deviation of the mean (SD). After 72 hr, a total of 68.4 +/- 74.4 eggs (normal and abnormal) were enumerated in the worm cultures containing 1% DMSO, 73.2 +/- 46.4 eggs (normal and abnormal) were observed in the cultures containing 10 μM 7-keto-sempervirol and zero eggs were counted in the wells containing 100 μM 7-keto-sempervirol. Student’s t-test indicates that a significant difference exists between the percentage of abnormal eggs laid between the 1% v/v DMSO and 10 μM 7-keto-sempervirol treatments (*p* = 0.01). B) Fluorescent images of representative eggs deposited *in vitro* demonstrate normal egg architecture in the DMSO control wells (DMSO control) and abnormal egg architecture in the 10 μM 7-keto-sempervirol wells (7-keto-sempervirol).

Laser scanning confocal microscopy (LSCM) of adult females cultured in the presence of 7-keto-sempervirol (100 μM for 24 hr) indicated the presence of irregularly shaped *in utero* eggs (Fig. 5). When compared to control eggs (parasites treated with 1% v/v DMSO, Fig. 5A), these abnormal eggs lacked regular autofluorescence as well as fully formed eggshells and were missing the characteristic lateral spines indicative of the species (Fig. 5B). Due to these phenotypic deficiencies in egg development, the effect that 7-keto-sempervirol had on *in vitro* schistosome oviposition was also assessed (Fig. 6). Here, 7-keto-sempervirol induced a concentration-dependent (100 μM > 10 μM) ability to inhibit the deposition of phenotypically normal schistosome eggs with a complete lack of oviposition observed in wells containing the highest amount of compound (100 μM) (Fig. 6A). When compared to control wells (schistosomes co-cultured with 1% v/v DMSO), eggs deposited in wells containing 7-keto-sempervirol (10 μM) displayed a range of abnormal phenotypes (Fig. 6B) similar to those observed *in utero* (Fig. 5B). These phenotypes included non-oval shapes, lack of lateral spines and irregular autofluorescence.

### Anti-*Fasciola* 7-keto-sempervirol activities

**Fig 7:**
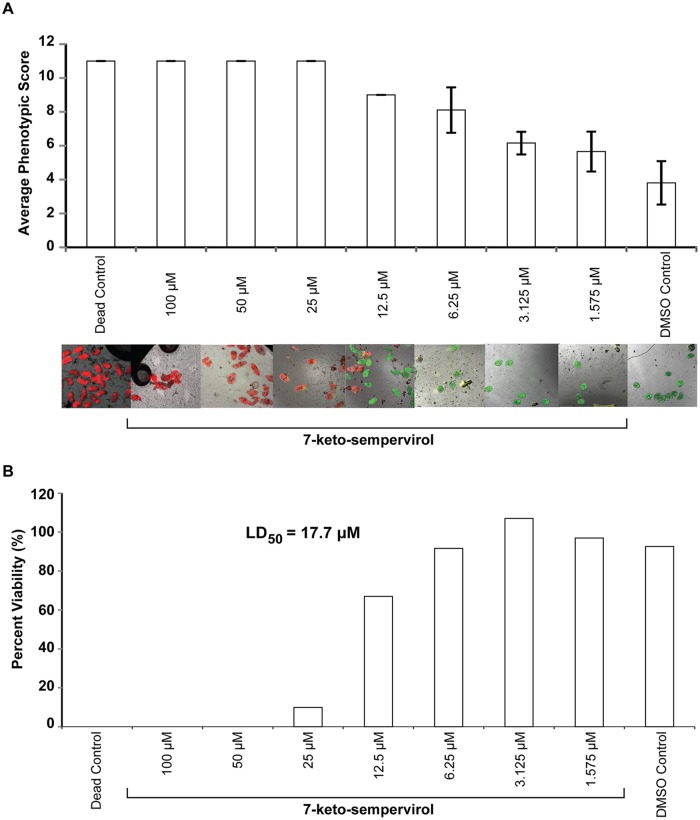
The diterpenoid 7-keto-sempervirol displays lethal activity against *Fasciola hepatica* newly excysted juveniles (NEJs). Complementary phenotypic-based [31,32] and fluorescent-mediated tests [25] were used to assess NEJ viability. A) The phenotypic assay involved co-culturing 20 NEJs (96-well plate format) with a 50% dilution series of 7-keto-sempervirol (100, 50, 25, 12.5, 6.25, 3.125, 1.563 μM) at 37°C and 5% CO_2_ for 24 hr. Mean phenotypic score values (11 = severely affected, 0 = not affected) are indicated by bar charts (n = 20 individuals scored/experimental treatment) with error bars representing the standard deviation of the mean (SD). A collection of fluorescent high content images (10 x) was obtained similar to those collected for schistosomula (Fig. 2). B) The fluorescent assay utilised the HFB [25] and co-cultured 100 NEJs/experimental treatment (96-well plate format) with the same dilution series of 7-keto-sempervirol used in the phenotypic-based test. Each concentration was converted into corresponding Log_10_ values and the percentage viability was transformed into probit values to create a dose-response curve. An LD_50_ of 17.7 μM was calculated from this dose-response curve. One representative HFB is illustrated here. Dead control = NEJs co-cultured with 70 μM auranofin. DMSO control = NEJs co-cultured with 1% v/v DMSO.

**Fig 8:**
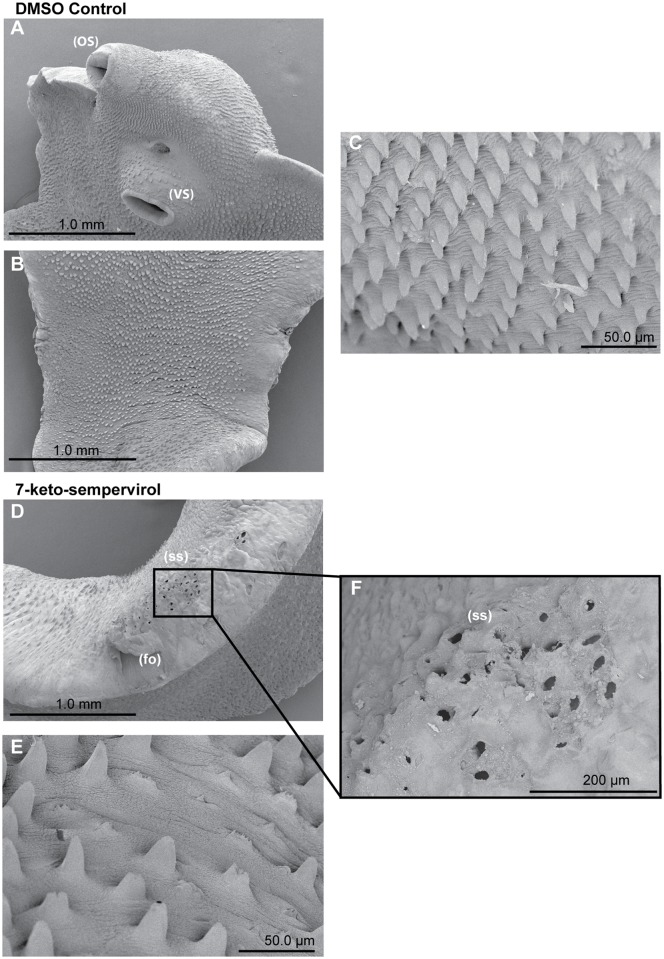
The diterpenoid 7-keto-sempervirol induces damage, shortening and loss of adult *Fasciola hepatica* tegumental spines. SEM images of adult *F*. *hepatica* 48 hr post-treatment with culture media containing DMSO (1% v/v) or 100 μM 7-keto-sempervirol. A) Normal fluke ventral surface architecture including oral sucker (OS) and ventral sucker (VS) is unaffected after cultivation with culture media containing 1% (v/v) DMSO. B) Fluke dorsal surface architecture is also unaffected after cultivation with culture media containing 1% (v/v) DMSO. C) Fluke ventral spine phenotype after cultivation with culture media containing 1% (v/v) DMSO. D) Dorsal spine sockets (ss) and folding (fo) after cultivation with 100 μM 7-keto-sempervirol. E) Ventral spines after cultivation with 100 μM 7-keto-sempervirol. F) Higher magnification of (ss) region outlined by black box in (D).

To assess 7-keto-sempervirol’s activity on *F*. *hepatica* NEJs, two complementary methodologies were employed (Fig. 7). The first methodology, using well-established motility and phenotypic metrics [31,32], indicated that 7-keto-sempervirol induced a negative concentration-dependent effect on NEJ movement and viability (Fig. 7A). This finding was supported by fluorescent microscopy of NEJs co-stained with the discriminatory viability dyes FDA and PI (Fig. 7A). The second methodology, using the HFB as a more objective method for determining NEJ viability [25], confirmed this concentration-dependent effect and established an LD_50_ of 17.7 μM for 7-keto-sempervirol against *F*. *hepatica* NEJs (Fig. 7B). When compared to the mammalian HepG2 cell line (S1 Fig.), 7-keto-sempervirol displayed an anti-NEJ therapeutic index of 4.5. To identify whether 7-keto-sempervirol also affected the surface integrity of *F*. *hepatica* adults, similar to *S*. *mansoni* (Fig. 4), SEM analyses were performed on adult liver flukes co-cultured with this diterpenoid (Fig. 8). Here, in comparison to control flukes incubated with 1% (v/v) DMSO (Fig. 8A-C), prolonged (48 hr) exposure to 7-keto-sempervirol (50 μM) induced substantial spine shortening and spine loss that was apparent on both dorsal and ventral sides of the organism (Fig. 8D-F).

## Discussion

Current control efforts aimed at reducing disease prevalence of schistosomiasis and fascioliasis are predominantly based on mono-chemotherapy administration of praziquantel (PZQ) and triclabendazole (TCBZ), respectively. Nevertheless, worries about the development of PZQ resistant blood flukes and the spread of TCBZ resistant liver flukes coupled to the slow progression of immunoprophylactic vaccines, have notably increased the number of new anthelmintic drug discovery projects initiated throughout the last decade. While our efforts in this area have successfully leveraged interdisciplinary techniques to identify drug targets [38–42] and to develop high-throughput drug screening methodologies [25], we have only recently begun characterising the detailed anthelmintic activities of defined chemical entities. Here, we report the findings from one such investigation and demonstrate that a diterpenoid (7-keto-sempervirol) derived from *Lycium chinense* has key properties useful in the development of a broad-spectrum agent active against both blood and liver flukes.

First amongst the examined properties was the ability of 7-keto-sempervirol to kill, in a concentration dependent manner, both *S*. *mansoni* schistosomula (Fig. 2) and *F*. *hepatica* newly excysted juveniles (NEJs) (Fig. 7) during *in vitro* culture. While this anti-larval effect was not overly potent (LD_50_ = 17.7 μM for schistosomula and LD_50_ = 19.1 μM for NEJs), it was selective (therapeutic index = 4.2–4.5) and reproducible (e.g. two independent measurements of NEJ viability provided confirmatory results, Fig. 7). Importantly, this moderate anti-larval effect translated into pronounced phenotypic abnormalities associated with the tegumental surface of both fluke species (Figs. 4 and 8). Here, and similar to artemisinin’s effect on *S*. *mansoni* [7] and artemether/artesunate’s effect on *F*. *hepatica* [43], 7-keto-sempervirol induced tubercle swelling, tegumental breaches and spine loss in both flukes. This degree of surface damage (also seen in [9,33,44]), if replicated *in vivo*, would severely compromise the barrier function of this protective layer [45] and negatively affect the ability of both flukes to remain in hostile definitive host environments (blood and bile). Whether 7-keto-sempervirol’s ability to disrupt fluke surface architecture is similar in action to the protonophoric properties of membrane-disruptive, plant-derived quinones (anti-tumour derived diterpenoids) [46] is currently unknown, but is consistent with our observations. Further work is required to develop this line of investigation.

Another interesting property of 7-keto-sempervirol is its ability to progressively paralyse adult schistosomes during *in vitro* culture (Fig. 3). This feature, in addition to tegumental alterations, is similar to the effects induced by praziquantel [47] treatment of adult schistosomes and suggests that this diterpenoid may also perturb calcium homeostasis [3]. Interestingly, the antifungal activities of two related mono-terpenoid phenols (carvacrol and thymol) also involve dis-regulation of intracellular calcium balances and suggest a common mechanism of action amongst terpenes [48] and praziquantel. In support of this, treatment of *Saccharomyces cerevisiae* with either carvacrol or thymol leads to the differential expression of similar gene categories as those found in praziquantel treated schistosomes [47]. These include genes associated with drug transportation across membranes, ribosomal biogenesis, autophagy, heat shock responses, rRNA processing, tRNA processing and pyrimidine metabolism [48]. Whether 7-keto-sempervirol is capable of differentially regulating a similar repertoire of schistosome gene products is currently unknown, but the time-dependent paralysis of adults co-cultured with this diterpenoid *in vitro* would suggest that this hypothesis is likely. Gender specific transcriptomic responses to 7-keto-sempervirol may also exist, similar to those induced by praziquantel [47], and could explain the differential sensitivity and hyperactivity observed in females (but not males) at 24 hr post treatment (Fig. 3). While 7-keto-sempervirol/adult *F*. *hepatica* co-cultures were not kinetically studied in this investigation, it would be useful to ascertain in future experiments if the diterpenoid-induced, time-dependent paralysis of dioecious blood flukes translates to hermaphroditic liver flukes.

A final anthelmintic property examined for 7-keto-sempervirol, based on its established capacity to damage surface membranes (Figs. 4 & 8) and impede schistosome motility (Fig. 3), was its potential to affect egg production during *in vitro* cultures (Figs. 5 & 6). As oviposition is required for schistosome lifecycle transmission and is responsible for chronic definitive host immunopathology, identification and progression of compounds that inhibit this process (even if they do not kill the parasite) are likely to benefit schistosomiasis control. Interestingly, the concentration-dependent (100 μM > 10 μM), 7-keto-sempervirol mediated, inhibition of phenotypically-normal egg maturation (Fig. 5 & 6B) and egg production (Fig. 6A) observed here are strikingly similar to those findings reported for schistosome pairs co-cultured with kojic acid [41]. Kojic acid exerts its effects on eggshell sclerotisation (hardening or tanning) and schistosome oviposition by inhibiting the phenol oxidase activities of *S*. *mansoni* tyrosinases 1 and 2 (SmTYR1/SmTYR2). Whether 7-keto-sempervirol also targets the phenol oxidase activity of SmTYR1/SmTYR2 is currently unknown. However, as egg maturation, sclerotisation and oviposition are complex processes involving cytosine methylation [40], tyrosine kinase-mediated phosphorylation [49] serine/threonine kinase-mediated phosphorylation [50], fatty acid oxidation [51] and TGF-beta signalling [52], 7-keto-sempervirol could theoretically act upon any component of these (or other) diverse biological processes. Limitations in obtaining sufficient quantities of adult *F*. *hepatica* prohibited the assessment of 7-keto-sempervirol’s effect on liver fluke egg production. Nevertheless, as the process of eggshell formation is thought to occur via similar mechanisms in both schistosomes and liver flukes [53], it is likely that 7-keto-sempervirol will also inhibit *F*. *hepatica* oviposition.

Here, we provide complementary evidence that supports the further progression of 7-keto-sempervirol as a dual anthelmintic against *S*. *mansoni* and *F*. *hepatica* parasites. Although these findings add value to the growing medicinal properties described for diterpenoids [54–56] and expand upon their anti-schistosomal chemoprophylactic characteristics [21,22], improving their selective potency is a necessary next step in their development as wide-acting anthelmintics. Whilst defining a specific mechanism of 7-keto-sempervirol action and experimental animal model verification was beyond the scope of this investigation, our results suggest that membrane biogenesis/maintenance and calcium homeostasis/stress are likely contributing to the diverse *in vitro* phenotypes observed in both fluke species. Activity against both larvae and adult fluke stages broaden the window of therapeutic opportunity and, if replicable *in vivo*, would provide a useful alternative to currently used anthelmintics within the biomedical and animal health landscapes.

## Supporting Information

S1 Fig 7-keto-sempervirol / human HepG2 cell titration for determination of LD_50_ concentration. (PDF)

S2 Fig Phenotypes of adult male and female schistosomes co-cultivated with DMSO for 24–72 hr are not significantly different. (PDF)

S3 Fig Adult schistosome phenotypes are not significantly affected by co-culture with 10 μM 7-keto-sempervirol. (PDF)
