# Supplementary figures and images for: The Diterpenoid 7-Keto-Sempervirol, Derived from Lycium chinense, Displays Anthelmintic Activity against both Schistosoma mansoni and Fasciola hepatica
Source: PLoS Negl Trop Dis. 2015 Mar 13;9(3):e0003604. doi: 10.1371/journal.pntd.0003604 (PMC4358835; doi:10.1371/journal.pntd.0003604)

## HepG2 cell line / 7-keto sempervirol titration

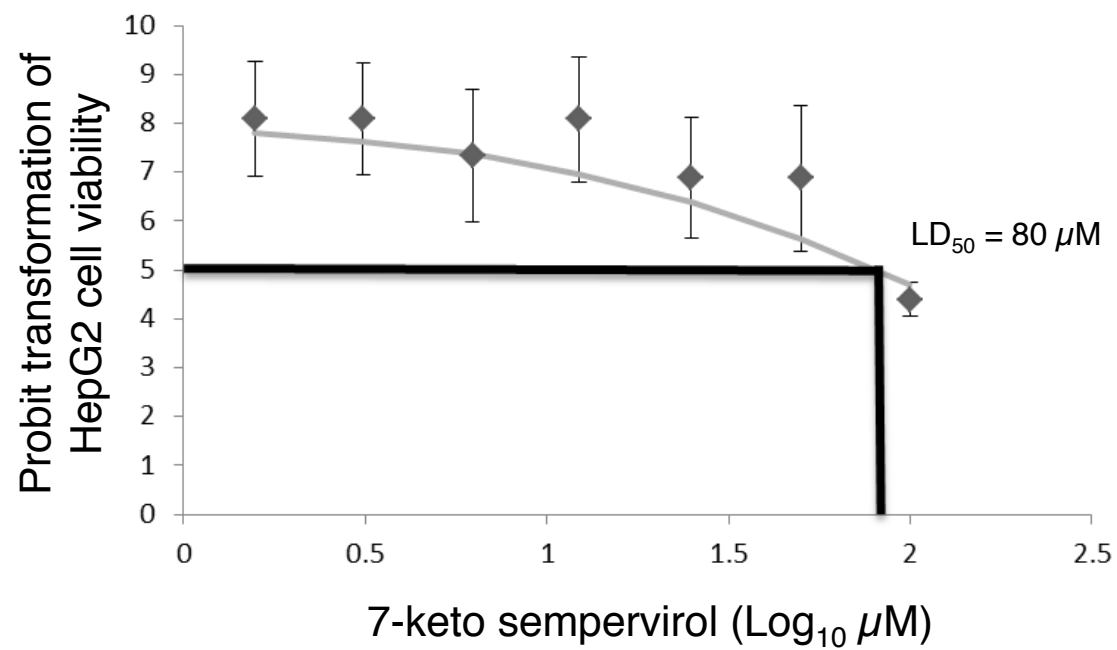

Supplement: S1 Fig — (PDF) [file pntd.0003604.s001.pdf]

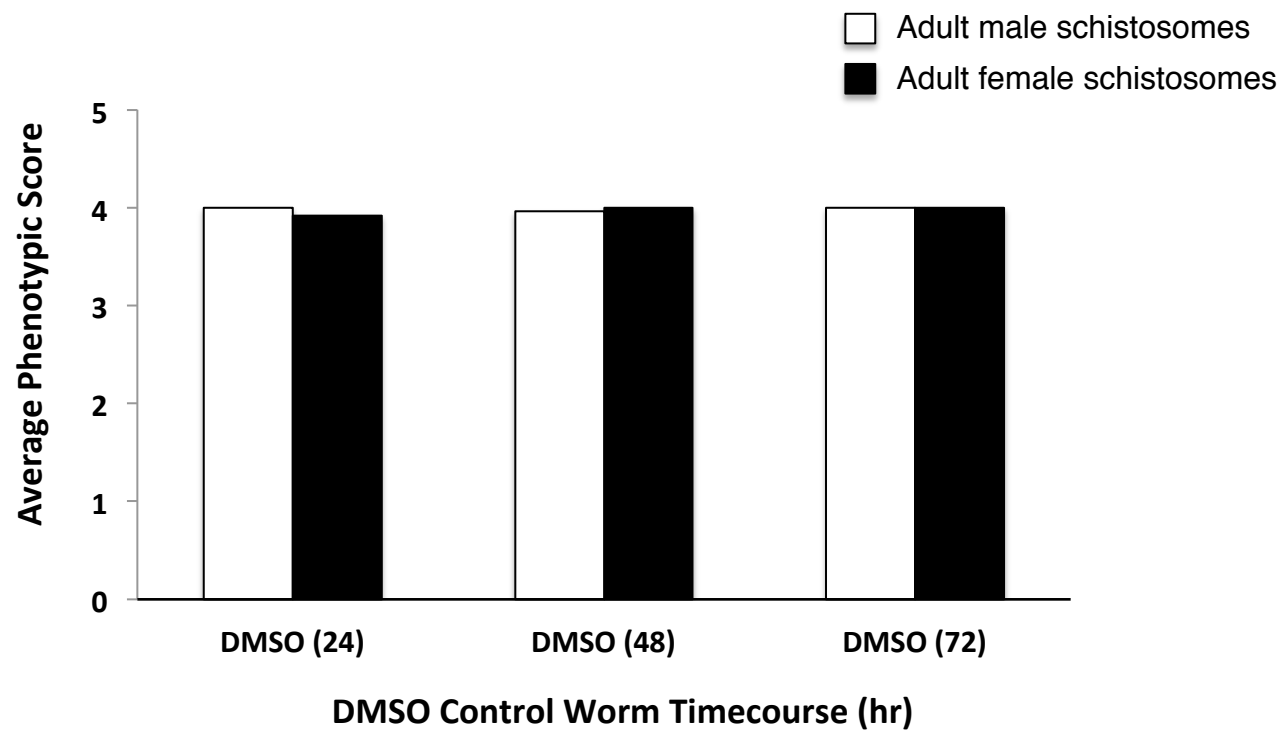

Supplement: S2 Fig — (PDF) [file pntd.0003604.s002.pdf]

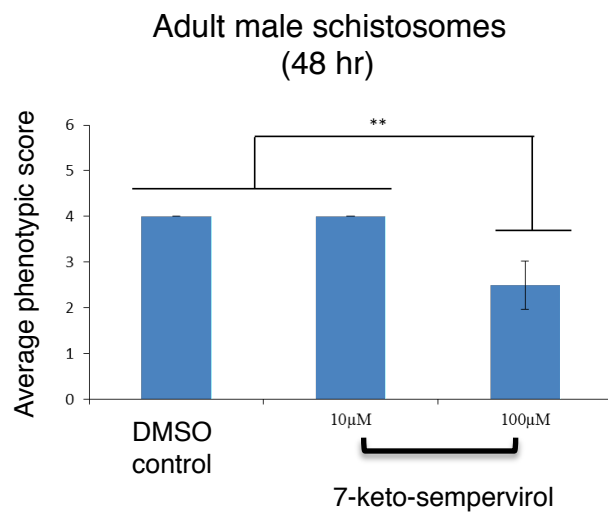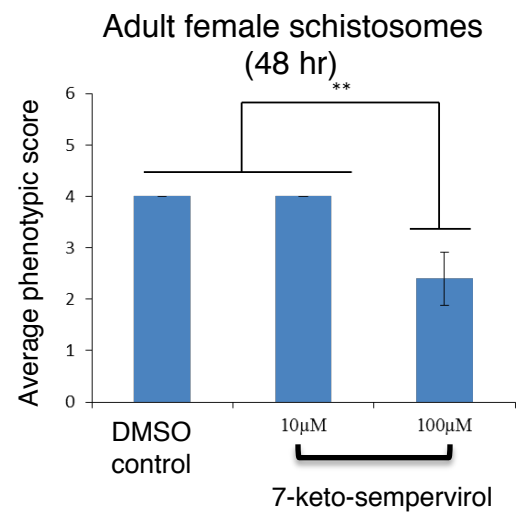

Supplement: S3 Fig — (PDF) [file pntd.0003604.s003.pdf]
